# Diphtheritic Poison

**Published:** 1879-09

**Authors:** 


### THE DIPHTHERITIC POISON.

A singular instance of the vitality of the poison of diphtheria is reported in the *Vrats Chebnyia Vedomosti.* A gentleman in the South of Russia, four years ago, lost a boy from diphtheria. A family vault having recently been constructed, the coffin of the boy was transferred thither.

Before it was lowered down into the vault, the father wished to look at the body, having entertained a suspicion that the child had been buried alive.

An opening was accordingly made in the lid of the coffin, and the whole family, including five children, looked in. The next day *all* of the children were ill with diphtheria, and one of them has since died.

